# Excessive glucocorticoids combined with RANKL promote the differentiation of bone marrow macrophages (BMM) into osteoclasts and accelerate the progression of osteoporosis by activating the SYK/SHP2/NF-κB signaling pathway

**DOI:** 10.18632/aging.206084

**Published:** 2024-08-27

**Authors:** Hao Dong, Xiaocong Liu, Jiqiang Duan, Jing Zhang, Hao Liu, Tiehui Shen

**Affiliations:** 1 West Campus of Zibo Central Hospital, Zibo, Shandong, China; 2 Zibo Central Hospital, Zibo, Shandong, China

**Keywords:** osteoporosis, glucocorticoids, BMM, RANKL, SYK/SHP2/NF-κB signaling pathway

## Abstract

The primary objective of this study was to explore the extensive implications and complex molecular interactions arising from the confluence of excessive glucocorticoids and RANKL on the differentiation process of BMM into osteoclasts, profoundly impacting osteoporosis development. The methodology encompassed X-ray analysis and HE staining for evaluating bone loss in mice, while immunohistochemical staining was utilized to observe phosphorylated SHP2 (p-SHP2) expression. The assessment of several phosphorylated and total protein expression levels, including NF-κB, SHP2, SYK, JAK2, TAK1, NFATC1, c-fos, and Cathepsin K, was conducted via Western blotting. Additional experiments, involving CCK8 and monoclonal proliferation assays, were undertaken to determine BMM proliferation capacity. Immunofluorescence staining facilitated the quantification of TRAP fluorescence intensity. *In vivo* analysis revealed that glucocorticoid surplus triggers SHP2 signaling pathway activation, accelerating osteoporosis progression. Western blot results demonstrated that SHP2 inhibition could decrease the expression of specific proteins such as p-NF-κB and p-SHP2, with minimal effects on p-SYK levels. *In vitro* findings indicated that glucocorticoid and RANKL interaction activates the SHP2 pathway through NF-κB and SYK pathways, enhancing expressions of p-JAK2, p-TAK1, NFATC1, c-fos, and Cathepsin K, thereby promoting BMM to osteoclast transformation.

Conclusion: Excessive glucocorticoids and RANKL interaction advance osteoclast differentiation from BMM by activating the SYK/SHP2/NF-κB signaling pathway, expediting osteoporosis progression.

## INTRODUCTION

Osteoporosis in the elderly (SOP) is an age-related disorder [1]. Elderly osteoporosis accounts for 20% of primary osteoporosis cases [2]. Factors such as Vitamin D deficiency, hormonal imbalance, protein-energy malnutrition, and dysfunction of the neuromuscular system, among others, may accelerate the progression of osteoporosis in the elderly [3]. In osteoporosis, the dynamics between osteoblasts and osteoclasts are altered. Osteoblasts, responsible for bone tissue formation, exhibit reduced activity, leading to a decreased rate of new bone tissue formation. Conversely, osteoclasts, responsible for bone resorption and degradation, increase in activity, resulting in accelerated bone resorption [4, 5].

Ovariectomy is frequently utilized as an animal model for osteoporosis and is also related to aging [6]. Post-ovariectomy, there is a notable increase in cognitive impairments and a significantly higher risk of Alzheimer’s disease [7]. Significant degenerative changes are detected in the skeletal muscle, musculotendinous junctions, tendons, tendon-bone interface, and periosteum post-ovariectomy [8]; cardiovascular aging is accelerated [9]. Ovariectomy leads to repercussions in the female reproductive system, subsequently elevating glucocorticoid levels [10]. Glucocorticoids, a class of synthetic steroid hormones, inhibit bone formation and promote bone resorption, thereby facilitating the development of osteoporosis. Long-term use of corticosteroids leads to osteoporosis, significantly increasing morbidity and mortality [11]. Cumulative corticosteroid doses greater than 10 g, age over 50, and pre-existing low bone mass are risk factors for corticosteroid-induced osteoporosis [12]. Glucocorticoids exert their anti-inflammatory effects by inhibiting the activation of NF-κB and subsequent expression of inflammation-related genes. This interaction between glucocorticoids and the NF-κB signaling pathway, especially involving the P65 subunit, a component of the transcription factor complex nuclear factor kappa B (NF-κB), is notable [13]. Osteoclasts, large multinucleated bone-resorbing cells derived from the fusion of monocyte/macrophage precursors, are thought to undergo apoptosis once resorption is complete [14]. Bone resorption and formation in bone tissue are continuously alternating processes. Bone resorption is carried out by multinucleated bone-resorbing cells from the monocyte/macrophage lineage, responsible for absorbing bone tissue. Conversely, bone formation is mediated by osteoblasts expressing the nuclear factor-kappa B ligand (RANKL), expressed as a cell membrane-associated cytokine. Osteoprotegerin (OPG) is a soluble RANKL interception receptor primarily produced by osteoblasts, inhibiting the interaction between RANKL and its receptor, thereby preventing bone resorption and the formation of bone-resorbing cells [15]. Monoclonal antibodies against RANKL can effectively inhibit the development and activity of osteoclasts [16]. RANKL’s mechanism of action mainly involves regulating bone metabolism by binding to the RANK receptor on bone marrow cells and osteoblasts. Studies have shown that the interaction between RANKL and the RANK receptor on bone marrow cells and macrophages promotes the production and activation of osteoclasts (bone-resorbing cells), leading to an increase in bone mineral substance and bone loss. Apart from directly promoting bone resorption, RANKL also indirectly inhibits bone formation by interfering with the differentiation of osteoblasts on bone marrow cells, further affecting bone healing and metabolism post-fracture [17, 18]. In osteoporosis, BMM (bone marrow macrophages) generally refers to macrophages present in the bone marrow. These cells play a key role in regulating bone metabolism and homeostasis. Studies indicate that bone marrow macrophages participate in critical processes in osteoporosis, including bone resorption during pathological processes. They promote the activation of osteoclasts by releasing cytokines and pro-inflammatory factors related to bone resorption, leading to bone loss [19, 20]. BMMs can differentiate directly into osteoclasts under M-CSF stimulation [21], with multiple signaling pathways playing a role. The PI3K/AKT pathway mediated by ATF4 can directly participate; elevated expressions of c-Fos and NFATc1 can also promote the generation of osteoclasts; the MEK/ERK, p38 MAPK, and JNK pathways inhibit the differentiation of bone marrow macrophages induced by RANKL [22]. In the skeleton, SYK is considered to be involved in regulating the bone resorption process, the cell-mediated loss of bone tissue, such as that caused by macrophages. SYK may influence the balance of bone remodeling by regulating the activity of macrophages and other skeletal cells, thereby affecting bone density and the development of osteoporosis [23, 24].

SHP2 plays a significant role in bone development and cartilage homeostasis, influencing the transdifferentiation of hypertrophic chondrocytes into osteoblasts and providing insights into the pathogenesis and potential treatments for skeletal diseases, such as osteoporosis and osteopenia [25]. SHP2 negatively regulates osteoblast differentiation through the MEK2 and AKT2 signaling pathways [26]. SHP2 regulates osteoclast generation by promoting the fusion of pre-osteoclasts [27]. NF-κB influences the development of osteoporosis in the skeletal system by regulating inflammatory responses, proliferation and differentiation of bone cells, and bone remodeling. Upregulation of the NF-κB pathway inhibits osteoblast formation and promotes osteoclast differentiation, increasing the risk of osteoporosis in rats [28]. Glucocorticoid-induced osteoporosis in neonates can be prevented by SIRT1 and NF-κB [29]. By regulating the NF-κB and p38 signaling pathways, osteoclast generation is inhibited, thereby alleviating postmenopausal osteoporosis [30].

Therefore, glucocorticoids can stimulate an increase in P65, further promoting an increase in SHP2 levels. Another pathway is RANKL stimulation of SYK phosphorylation, promoting an increase in SHP2 levels. The increase in SHP2 levels can promote an increase in P-JAK2 and P-TAK1. Through *in vivo* and *in vitro* experiments, the effects and molecular mechanisms of glucocorticoid overuse and RANKL on BMM differentiation into osteoclasts and the progression of osteoporosis have been explored. This study will provide new therapeutic targets and methods for the treatment of osteoporosis.

## METHODS

### Construction of SHP2 silencing vector

The coding sequence (CDS) region of SHP2 was obtained from the NCBI database by searching for its transcript number. The pSIH1-H1-copGFP-T2A-Puro vector was used as the backbone for designing specific shRNAs through the GPP Web Portal to knock down the SHP2 gene in mice. The synthesized shRNAs were annealed and cloned into the pLKO_005 vector. This construct was then introduced into the pHAGE-CD19 vector equipped with a macrophage-specific synthetic promoter through PCR, enzyme digestion, and T4 ligase linking. The pHAGE-CD19, psPAX2, and pMD2.G plasmids were co-transfected into 293T cells using Lipo8000. The medium was changed to DMEM containing 10% FBS the next day. After 72 hours, the supernatant was collected, and centrifuged at 3000 rpm for 15 minutes at 4°C, and the viral fluid was stored at −80°C.

### Experimental animal model

Twenty-four SPF-grade female BABL/C mice were obtained from Henan Scot-Bios Biotechnology Co., Ltd. Before the experiments, the animals were acclimatized for a week in a laboratory with a temperature of 18–22°C, the humidity of 65 ± 5%, good ventilation, and a quiet environment. They had free access to water and were fed standard feed. A 3% solution of sodium pentobarbital (40 mg/kg) was used for intraperitoneal anesthesia. After the mice were anesthetized and immobilized, their abdomens were shaved below the xiphoid process. The surgical site was disinfected with 75% alcohol, and covered with a sterile drape, and a 2 cm longitudinal incision was made below the abdominal midline to expose the abdominal cavity. The ovaries were located along the uterine horns and carefully removed. The fallopian tubes, surrounding blood vessels, and adipose tissues were ligated with catgut. Post-surgery, the mice were placed in sawdust-lined cages and received daily intramuscular injections of penicillin for five days to prevent infection. BMMs transfected with SHP2-NC and shRNA-SHP2, and BMSCs were injected into mice via the tail vein. Mice were divided into non-SOP, SOP, and SOP+ shRNA-SHP2 groups. An X-ray examination was conducted to detect osteoporosis. Mice were injected weekly with the virus vector carrying the SHP2 silencing fragment (5 × 10^11^ vg/mouse) via the tail vein. After four weeks of continuous injection of SHP2 lentivirus, an X-ray examination was performed to detect osteoporosis. Then, the mice were euthanized with isoflurane, and femur tissue samples were collected.

### Primary isolation of BMM cells

Sterile techniques were required during and after the isolation of bone marrow cells. All instruments were carefully cleaned with ethanol. Bone marrow was collected under a laminar flow hood after euthanizing mice with isoflurane. The abdomen and hind limbs were disinfected with 70% ethanol. An incision was made along the abdominal midline to expose the hind legs. All muscle tissues were removed from the bones with scissors. The ends of the bones were cut to release them. In a mortar, the bones were crushed with 5 mL of lymphocyte culture medium supplemented with 20 mM HEPES. Alternatively, femurs and tibias were separated by cutting at the knee joint. Bone marrow was flushed with lymphocyte culture medium using a 5 mL syringe and a 25-gauge needle. Bone marrow cells were pipetted up and down to form a single-cell suspension. Cells were filtered through a cell strainer, and the strainer was rinsed with an additional 5 mL of lymphocyte culture medium. Bone marrow cell counts were determined using a hemocytometer. The concentration was adjusted to 2 × 10^6^ cells/mL in a BMM culture medium.

Cells were cultured in 15 cm culture dishes with 25 mL of medium, differentiated in a humidified incubator at 37°C with 5% CO_2_. Cells were washed with PBS every 2–3 days, and a fresh BMM culture medium was added. Cells from 15 cm diameter BMM culture dishes were resuspended directly using a pipette and then transferred to tubes.

### BMM cell transfection

A lymphocyte culture medium containing 20% FBS was prepared. An appropriate amount of medium was added to each well of a 12-well plate, 1.5 mL per well, and the plate was pre-incubated in a humidified incubator at 37°C, 5% CO_2_. The harvested BMMs were counted and centrifuged at 200 g for 10 minutes. The cell suspension was resuspended to a concentration of 1 × 10^6^ cells/100 μL (minimum 100 μL) of reagent, and an appropriate amount of lentivirus was added for cell transduction. 1–2 μg of shRNA SHP2 lentivirus was added to 1.5 mL tubes. The diluted cell suspension was transferred back to the prepared 12-well plate.

BMMs were co-induced with M-CSF (100 ng/ml) and RANKL to differentiate into osteoclasts. BMMs were treated with SYK inhibitor R406 and GR inhibitor Nenocorilant. BMMs were divided into control, SYK inhibitor, GR inhibitor, and SHP2-KD groups.

### Immunohistochemistry staining

Tissues were fixed in 10% neutral buffered formalin, embedded in paraffin, and sectioned into 4 μm slices using a microtome. Tissue sections were deparaffinized with xylene and ethanol, and antigen retrieval was performed using a citrate antigen retrieval solution. Endogenous peroxidase blocker was dropped on the tissue surface, followed by blocking with 5% goat serum. Sections were incubated overnight with P-SHP2 Rabbit mAb at 4°C, then incubated with enzyme-linked goat anti-rabbit IgG polymer at room temperature for 20 minutes. A prepared DAB chromogen solution was added to the tissue, covering the entire section. Staining was stopped when the reaction turned brownish-yellow. Sections were differentiated with hydrochloric acid alcohol, counterstained with hematoxylin, dehydrated through a graded series of alcohols, cleared with xylene, and mounted with neutral balsam. Images of tissue sections were captured using a Leica microscope imaging system. Staining was assessed using grayscale density analysis. Immunohistochemistry images were identified and analyzed using ImageJ Fiji (National Institutes of Health, NIH, USA) software to obtain AOD values. Statistical analysis was performed using GraphPad Prism9.0 statistical software.

### Hematoxylin and eosin (H&E) staining

After extracting the tissue, the tissue specimens were gradually immersed in ethanol solutions to dehydrate the specimen; cryosections were fixed for 30 seconds. After washing with water, the sections were stained with hematoxylin solution at 60°C for 60 seconds. The hematoxylin was then washed off with running water and 1% hydrochloric acid in ethanol for 3 seconds, followed by a brief rinse with water for 2 seconds. The sections were blued in ammonia water for 10 seconds and then rinsed with running water for 30 seconds. Eosin staining was performed for 60 seconds using a 0.5% eosin solution, followed by a brief rinse with distilled water. The sections were dehydrated in an ascending series of ethanol concentrations: 80% ethanol for 2 seconds, 95% ethanol for 2 seconds, and absolute ethanol for 2 seconds. The sections were cleared in xylene for 3 seconds, repeated twice, and then mounted with neutral balsam.

### Cell counting kit-8 (CCK-8) assay

100 μL of cell suspension was added to each well of a 96-well plate and pre-incubated in an incubator for 24 hours at 37°C and 5% CO_2_. Then, 10 μL of various concentrations of the test substance was added to each well. The plate was incubated for 12, 24, or 48 hours in the incubator. After incubation, 10 μL of CCK-8 solution was added to each well, being careful to avoid bubble formation as this can affect the optical density (OD) readings. The plate was further incubated for 4 hours in the incubator. The absorbance was measured at 450 nm using a microplate reader.

### Monoclonal formation experiment

Bone marrow macrophages (BMMs) were collected, digested with trypsin, counted, and cultured in an incubator at 37°C for about 2 weeks until visible cell colonies formed. The medium was then discarded, and the cells were washed three times with PBS, fixed with methanol for 15 minutes, air-dried, and stained with crystal violet for 30 minutes. After air-drying, the cells were scanned and photographed, and visible cell colonies were counted.

### Immunofluorescence staining

For immunofluorescence staining analysis, after washing, the sections were permeabilized with Triton-X and then blocked with goat serum for 30 minutes to prevent non-specific binding. The sections were incubated overnight with the primary antibody against TRAP. After washing with PBST, the sections were incubated at room temperature with Alexa Fluor-conjugated secondary antibody (1:400) for three hours. Finally, the sections were stained with DAPI and sealed with glycerol. Confocal microscopy was used to visualize and capture images of the stained sections.

### Western blotting

Samples were homogenized in RIPA lysis buffer. Cell proteins were collected using the same method. The total protein concentration was determined using a BCA Protein Assay Kit according to the manufacturer’s instructions. Proteins were separated by SDS-PAGE and transferred to a PVDF membrane. The membrane was blocked in 5% skim milk in TBS containing 0.1% Tween-20 at 37°C for 1 hour, then incubated overnight at 4°C with primary antibodies against p-NF-κB, p-SHP2, t-SHP2, p-SYK, p-JAK2, p-TAK1, NFATC1, c-fos, Cathepsin K, and GAPDH. The next day, the membrane was washed and incubated at 37°C for 1 hour with horseradish peroxidase-conjugated secondary antibodies, and visualized using Enhanced Chemiluminescence (ECL, BioRad, USA). ImageJ software (NIH, USA) was used to analyze the grayscale values of the target bands.

### Statistical analysis

Statistical analysis was performed using Graph Pad Prism software. Quantitative data were expressed as mean ± standard deviation. One-way ANOVA was used for comparisons among multiple groups, and the Tukey test was used for comparisons between two groups. A *P*-value < 0.05 was considered statistically significant.

## RESULTS

### Macrophage-specific knockout of SHP2 ameliorates osteoporosis in ovariectomized mice

**Figure 1:**
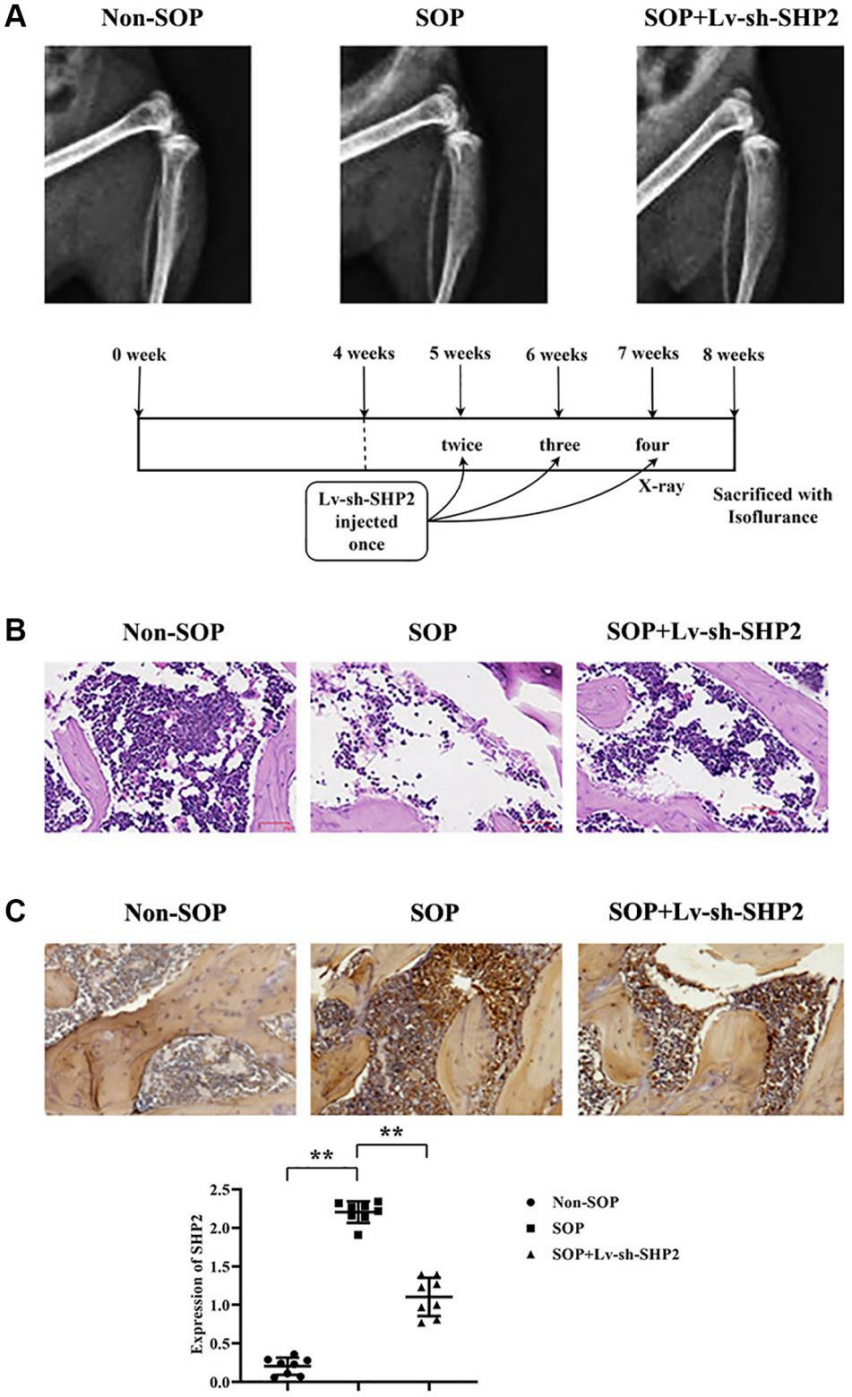
**Effects of macrophage-specific knockout of SHP2 on ovariectomy-induced osteoporosis.** (**A**) X-ray imaging was performed to assess bone density, bone quality, trabecular structure, and surface abnormalities in tibiae of mice from different experimental groups. SOP (ovariectomized) mice exhibited lower bone density, thinner bone quality, sparse trabeculae, and surface abnormalities compared to non-SOP mice. Conversely, SOP mice with macrophage-specific SHP2 knockdown (SOP+Lv-sh-SHP2) showed improved bone density, thicker bone quality, and preserved trabecular structure. (**B**) Hematoxylin and eosin (H&E) staining of vertebral sections revealed decreased cell numbers, enlarged gaps, and thinner trabeculae with fractures in SOP mice compared to non-SOP mice. However, SOP+Lv-sh-SHP2 mice displayed increased cell numbers and more continuous trabeculae. (**C**) Immunohistochemistry staining showed increased expression of phosphorylated SHP2 (p-SHP2) in SOP mice compared to non-SOP mice. Knockdown of SHP2 in macrophages resulted in decreased p-SHP2 expression, confirming successful knockdown. ^**^P < 0.01.

X-ray imaging demonstrated that, compared to the Non-SOP group, mice in the SOP group with ovariectomy exhibited significantly lower bone density, thinner bone quality, potentially more blurred edges, sparse bone trabeculae with trabeculae appearing sparse or disappeared, and occurrences of fractures or collapses in the tibia, along with abnormalities on the surface of the tibia. However, in the SOP+Lv-sh-SHP2 group of mice, where SHP2 was specifically knocked down in macrophages, there was a significant increase in bone density, thicker bone quality, clearer edges, and the trabeculae did not disappear, with no fractures or collapses observed on the tibia surface (Figure 1A).

Hematoxylin and eosin (H&E) staining results revealed a decrease in vertebral cell numbers, enlarged gaps, and thinner bone trabeculae with multiple fractures in SOP group mice compared to the non-SOP group. In contrast, mice in the SOP+Lv-sh-SHP2 group showed a notable increase in vertebral cell numbers and continuousness of the trabeculae compared to SOP group mice with ovariectomy. (Figure 1B).

Immunohistochemistry staining results indicated a significant increase in p-SHP2 expression in SOP group mice compared to the Non-SOP group. Meanwhile, p-SHP2 expression in the SOP+Lv-sh-SHP2 group was close to weak, suggesting a successful knockdown (Figure 1C).

### Macrophage-specific knockout of SHP2 in ovariectomized mice inhibits kinase activity and transcription factor levels

**Figure 2:**
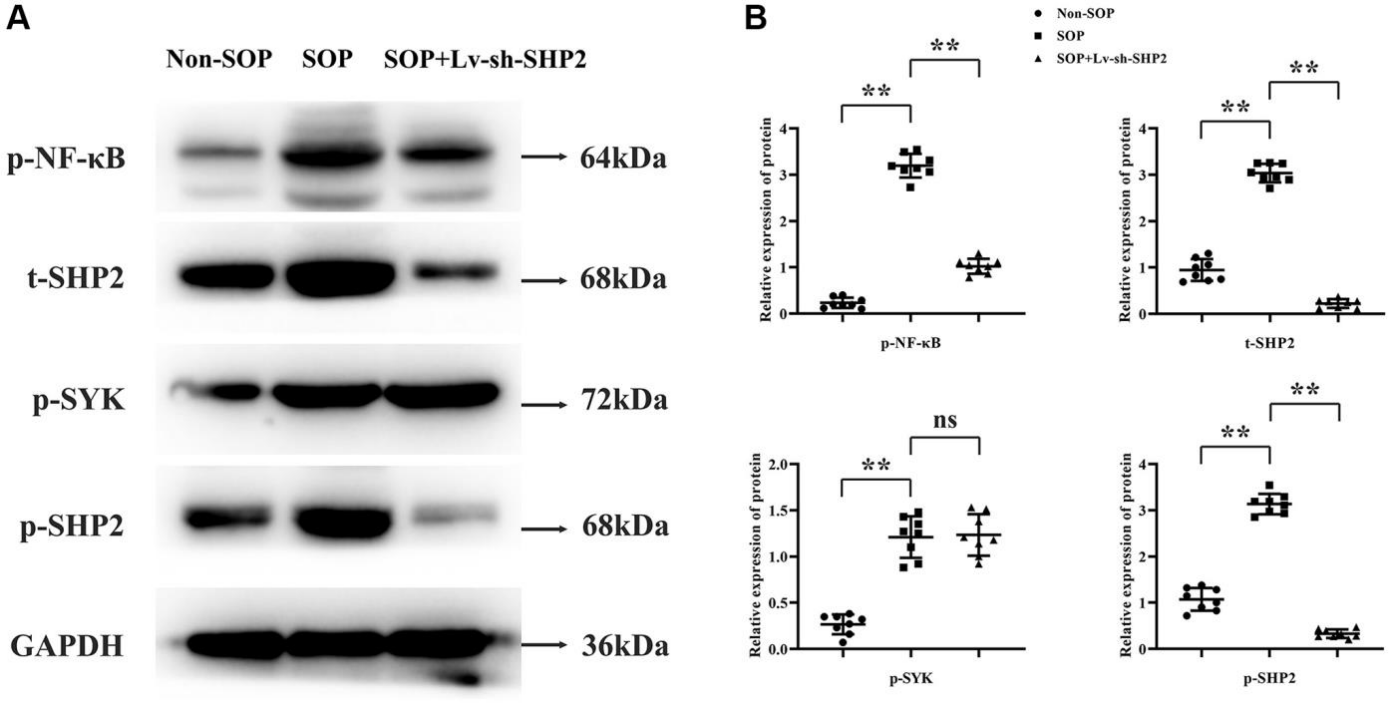
**Impact of macrophage-specific SHP2 knockout on kinase activity and transcription factors in ovariectomized mice.** (**A**, **B**) Western blot analysis was performed on tissue samples to evaluate the protein expression levels of phosphorylated NF-κB (p-NF-κB), spleen tyrosine kinase (p-SYK), and total and phosphorylated SHP2 (t-SHP2 and p-SHP2, respectively). SOP mice showed elevated levels of these proteins compared to non-SOP mice. In contrast, SOP mice with macrophage-specific SHP2 knockdown exhibited reduced levels of p-NF-κB, p-SHP2, and t-SHP2, with unchanged p-SYK levels. ^**^P < 0.01; ^ns^*P* > 0.05.

To investigate how macrophage-specific knockout of SHP2 affects the mechanisms of osteoporosis, we examined the protein expression levels of tyrosine kinase SYK (Spleen Tyrosine Kinase) and transcription factor NF-κB. Tissue from the cartilaginous sections was ground in liquid nitrogen, and total protein was extracted. Western blotting results showed that, compared to the Non-SOP group, the SOP group had significantly increased relative protein expression levels of p-NF-κB, p-SYK, p-SHP2, and t-SHP2. In comparison to the SOP group, the SOP+Lv-sh-SHP2 group exhibited significantly reduced levels of p-NF-κB, p-SHP2, and t-SHP2, while p-SYK levels remained unchanged, suggesting that SYK’s influence on osteoporosis might be upstream of the SHP2 pathway (Figure 2).

### Glucocorticoid receptor in conjunction with RANKL activates the SYK/SHP2/NF-κB signaling pathway to promote phosphorylation of JAK2 and TAK1

**Figure 3:**
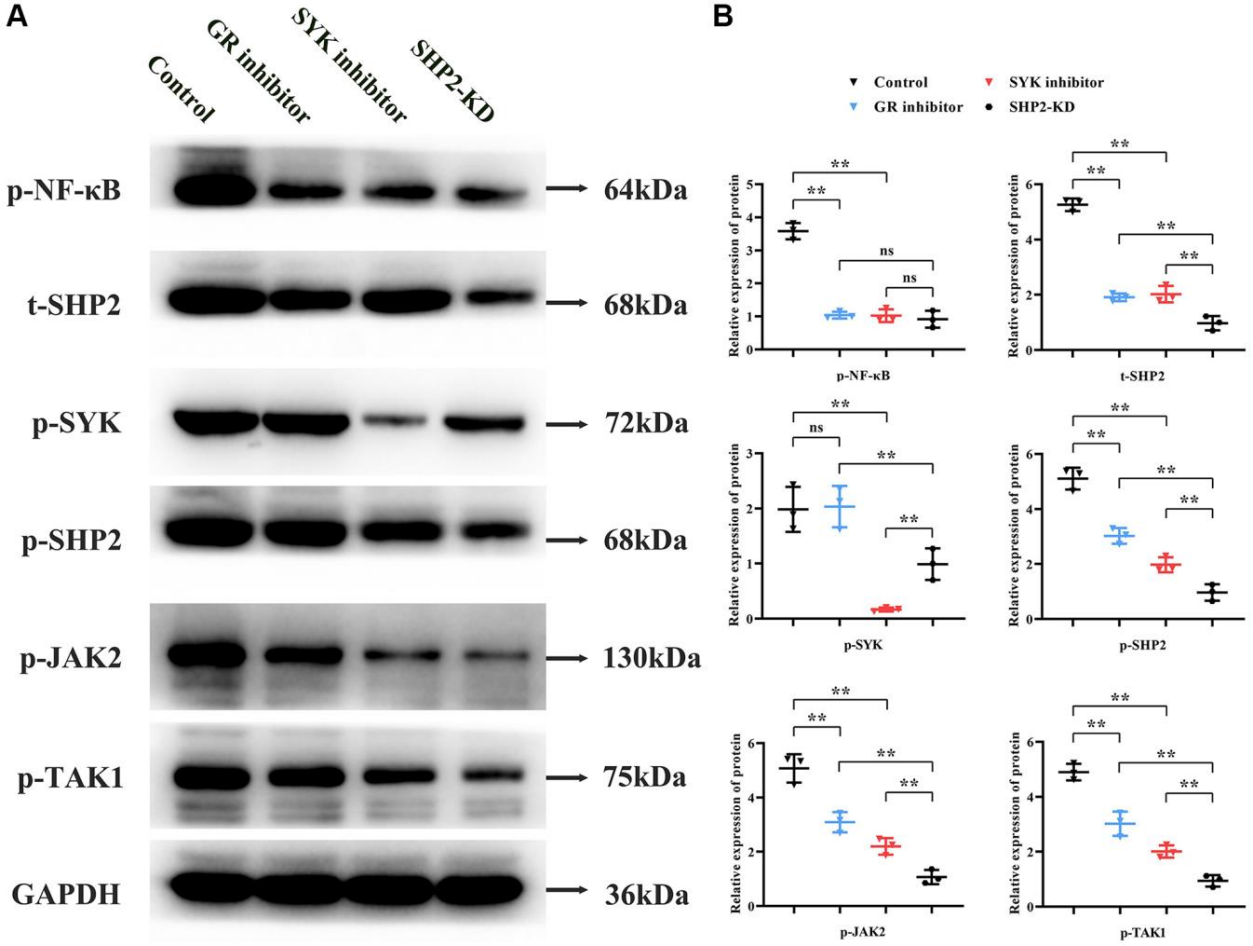
**Role of the SYK/SHP2/NF-κB signaling pathway in osteoporosis.** (**A**, **B**) Western blot analysis of primary cultured BMM cells treated with inhibitors targeting glucocorticoid receptor (GR), SYK, or SHP2 kinase domains (SHP2-KD) was performed. Inhibition of GR, SYK, or SHP2 resulted in decreased phosphorylation levels of NF-κB, SYK, SHP2, JAK2, and TAK1. Notably, SHP2-KD led to reduced p-SHP2, t-SHP2, p-JAK2, and p-TAK1 levels compared to SYK inhibition, suggesting SHP2’s downstream role in the pathway. ^**^P < 0.01; ^ns^*P* > 0.05.

Primary cultured BMM cells underwent total protein extraction followed by Western blot analysis. Compared to the Control group, the GR inhibitor group exhibited a significant reduction in relative protein expression levels of p-NF-κB, p-SHP2, t-SHP2, p-JAK2, and p-TAK1, with no significant change in p-SYK expression. The SYK inhibitor group showed a significant decrease in p-NF-κB, p-SYK, p-SHP2, t-SHP2, p-JAK2, and p-TAK1 levels compared to the Control group. Compared to the GR inhibitor group, the SHP2-KD group had similar levels of p-NF-κB but reduced expression of p-SYK, p-SHP2, t-SHP2, p-JAK2, and p-TAK1. Compared to the SYK inhibitor group, the SHP2-KD group had similar p-NF-κB levels, increased p-SYK expression, and decreased p-SHP2, t-SHP2, p-JAK2, and p-TAK1 levels (Figure 3).

### The glucocorticoid receptor in conjunction with RANKL activates the SYK signaling pathway to increase levels of NFATC1, c-fos, and cathepsin K, thus promoting proliferation and differentiation of BMMs into mature osteoclasts

**Figure 4:**
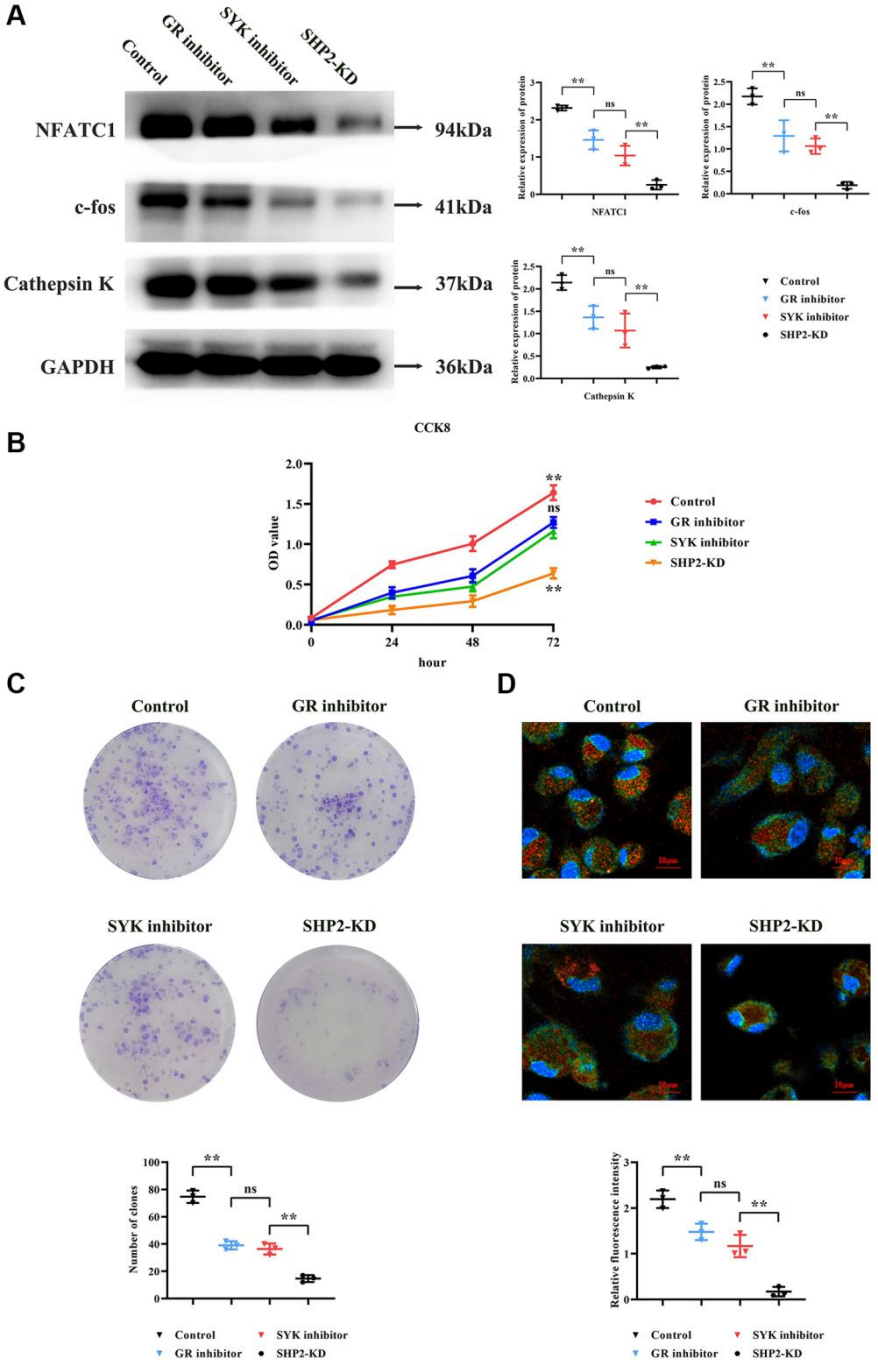
**Modulation of osteoclastogenesis by the SYK/SHP2/NF-κB pathway.** (**A**) Western blot analysis revealed decreased expression levels of NFATC1, c-fos, and Cathepsin K in GR inhibitor-treated BMM cells, with further reductions observed upon SYK inhibition and SHP2-KD. (**B**) Cell viability assay (CCK8) showed decreased optical density (OD) values in GR and SYK inhibitor-treated groups compared to controls, with a significant reduction in SHP2-KD cells. (**C**) Monoclonal formation assay demonstrated decreased colony formation in GR and SYK inhibitor-treated groups, with a further reduction in SHP2-KD cells. (**D**) Immunofluorescence staining for tartrate-resistant acid phosphatase (TRAP) indicated decreased fluorescence intensity in SHP2-KD, SYK inhibitor, and GR inhibitor-treated groups compared to controls, suggesting reduced presence of bone-resorbing cells. Abbreviations: SOP: Ovariectomized mice with osteoporosis; Non-SOP: Non-ovariectomized control mice; Lv-sh-SHP2: Lentivirus-mediated SHP2 knockdown; GR: Glucocorticoid receptor; BMM: Bone marrow-derived macrophages; OD: Optical density; NC: Negative control. ^**^P < 0.01; ^ns^*P* > 0.05.

Western blotting results revealed that, compared to the Control group, the GR inhibitor group showed a significant decrease in relative protein expression levels of NFATC1, c-fos, and Cathepsin K. Compared to the GR inhibitor group, the SYK inhibitor group experienced a further reduction in NFATC1, c-fos, and Cathepsin K levels. Compared to the SYK inhibitor group, the SHP2-KD group exhibited the lowest levels of NFATC1, c-fos, and Cathepsin K (Figure 4A).

CCK8 assay results indicated that at 72 hours, the OD values of both the GR inhibitor and SYK inhibitor groups were significantly lower than the Control group, with no statistical difference between the GR and SYK inhibitor groups. Compared to the SYK and GR inhibitor groups, the SHP2-KD group showed a significant reduction in OD values (Figure 4B).

Monoclonal formation experiment results showed that, compared to the Control group, the number of colonies formed in both the GR and SYK inhibitor groups was significantly reduced, with no noticeable difference between them. Compared to the SYK and GR inhibitor groups, the SHP2-KD group exhibited a significant reduction in colony formation (Figure 4C).

Immunofluorescence staining assessed the formation of bone-resorbing cells. TRAP (tartrate-resistant acid phosphatase) is a key marker for bone-resorbing cells, primarily found in osteoclasts and precursors of the monocyte/macrophage lineage. TRAP expression and activity significantly increase during the formation and activation of bone-resorbing cells. Results showed a significant reduction in relative TRAP fluorescence intensity in the SHP2-KD, SYK inhibitor, and GR inhibitor groups compared to the NC group. Compared to the SYK and GR inhibitor groups, the SHP2-KD group showed a significant reduction in TRAP fluorescence intensity, indicating a diminished presence of bone-resorbing cells (Figure 4D).

### The mechanism of SHP2 in osteoporosis

**Figure 5:**
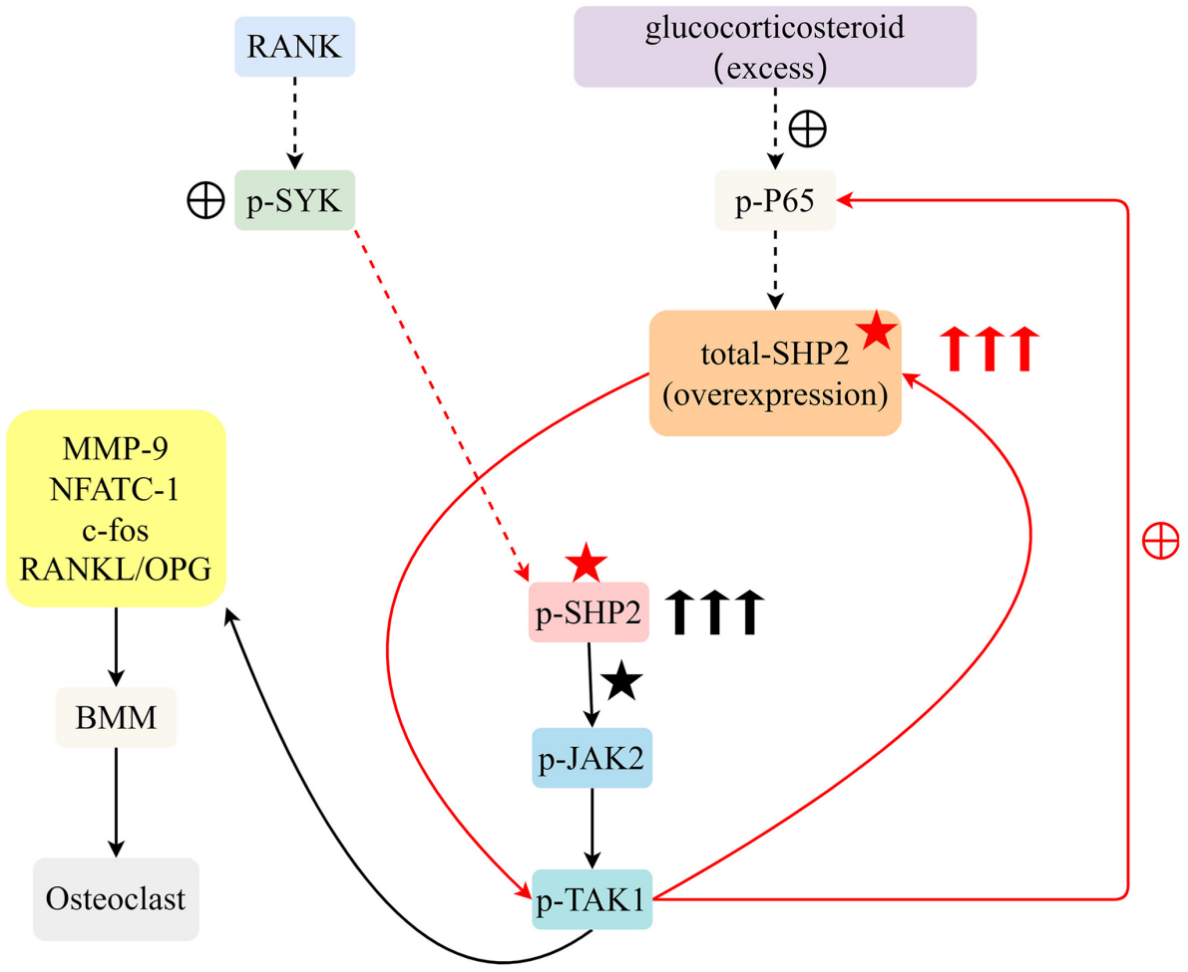
Excessive glucocorticoids and RANKL interaction advance osteoclast differentiation from BMM by activating the SYK/SHP2/NF-κB signaling pathway, expediting osteoporosis progression.

In the molecular signaling diagram, we observe a complex interaction of pathways that converge on the protein SHP2. This diagram illustrates how external stimuli such as glucocorticosteroids and RANKL (Receptor Activator of Nuclear Factor κB Ligand) initiate a cascade of phosphorylation events leading to an increase in certain proteins. Excess glucocorticosteroids stimulate the phosphorylation of P65, a subunit of the NF-κB (nuclear factor kappa-light-chain-enhancer of activated B cells) transcription factor. The phosphorylation of P65 is directly correlated with the upregulation of SHP2 (Src Homology 2-containing protein tyrosine Phosphatase 2) expression. Parallel to the action of glucocorticosteroids, RANKL is capable of promoting the phosphorylation of SYK (Spleen Tyrosine Kinase), which also contributes to the increase in SHP2 levels. The increase in total SHP2 leads to enhanced levels of phosphorylated SHP2 (p-SHP2), which in turn elevates the phosphorylation of JAK2 (Janus kinase 2) and TAK1 (TGF-beta activated kinase 1). The increase in phosphorylated JAK2 and TAK1 suggests an amplification of downstream signaling pathways that may lead to various cellular responses, which are not specified in the provided information but can be hypothesized based on known functions of JAK/STAT and MAPK pathways commonly associated with these kinases. The signaling pathway presented indicates a dual mechanism of SHP2 upregulation via glucocorticosteroids and RANKL pathways, which results in increased activity of JAK2 and TAK1, likely affecting cellular functions such as proliferation, differentiation, and survival (Figure 5).

## DISCUSSION

Osteoporosis is recognized as a pervasive and chronic malady that frequently intersects with the purview of orthopedic medicine. Due to the intensifying phenomenon of global demographic senescence, it has soared to preeminence as a predominant concern for public health across the world. Manifestations of this bone-weakening disease, ranging from fractures to chronic discomfort and skeletal anomalies, severely hamper an individual’s corporeal fortitude and overall life quality. These afflictions incite a surge in healthcare outlays. Within the orthopedic sphere, the exigency to probe the undercurrents of osteoporosis’ pathogenesis and to pinpoint prospective therapeutic interventions has crystallized into an imperative scientific quest [31].

Glucocorticoids wield their effect by curtailing osteoblast development and augmenting osteoclastic bone absorption, thus exacerbating osteoporosis risk with prolonged application. Such compounds actuate the NF-κB signaling axis, escalate SHP2 expression, and catalyze the phosphorylation of JAK2 and TAK1, culminating in osteoclast activation. In synergy with RANKL, glucocorticoids potentiate osteoclastogenesis via the SYK/SHP2/NF-κB cascade, hence hastening osteoporosis’ advancement. The induction of RANKL transcription by these steroids fosters osteoclast diversification, which culminates in amplified bone reabsorption. Additionally, glucocorticoids impede genes pivotal to osteogenesis, thereby stymieing bone genesis. Osteoclasts are now identified as the primary malefactors in devastating osseous disorders, including osteoporosis. The RANKL receptor activator’s role within the NF-κB signaling milieu is irrefutably central to the regulation of osteoblast genesis. RANKL’s hyperactive signaling promotes osteoblastic formation and bone resorption, intimating that curtailing the RANKL signaling trajectory may present an efficacious approach to osteoporosis remedy. Furthermore, potent doses of glucocorticoids, exemplified by dexamethasone, can precipitate the acceleration of osteoporosis and agitate osteoblast genesis. SHP2 has found links to skeletal diseases like Noonan syndrome, metachondromatosis, and osteoarthritis.

Elucidating SHP2’s role in bone remodeling and equilibrium maintenance is a convoluted task. Aberrant SHP2 levels can sway the differentiation and maturity of osteoblasts, osteoclasts, and chondrocytes. Concurrent mutations in SHP2 display repercussions on the immune, vascular, and neurological systems, which interplay with bone growth and remodeling [32]. Chlorogenic acid exhibits a preventative capacity against osteoporosis in ovariectomized rats via the SHP2/PI3K/Akt pathway, bolstering bone mineral density (BMD) and rectifying trabecular microarchitecture. Estrogen engages estrogen receptor α (ERα) to form a conglomerate with SHP2 and c-Src, mitigating c-Src’s activation and thus hampering osteoclastic bone resorptive actions [33]. Experimental observations in Noonan Syndrome (NS) murine models delineate SHP2’s advantageous impacts on osteoblast differentiation and osteoclastogenesis suppression. Moreover, bone mineral density (BMD) demonstrates a correlation with diminished muscle mass and a downward trend in IGF-1 levels [34–36]. In this line of investigation, SHP2’s regulatory effects inversely impede osteoblastic differentiation through the MEK2 and AKT2 signaling conduits, decelerating newfound osseous tissue formation. The involvement of SHP2 in the amalgamation of osteoblastic precursors aids in osteoblast composition. The augmented presence of SHP2 associates with the NF-κB pathway, which occupies a pivotal role in bone cell propagation, diversification, and structural reconfiguration. In rodent archetypes, an elevated NF-κB pathway represses osteoblast formation, engendering heightened osteoporosis susceptibility. SHP2 stimulates the amalgamation of osteoclastic progenitors, thereby orchestrating osteoclast production. In contrast, SHP2 reversely modulates osteoblastic differentiation through the MEK2 and AKT2 conveyances, thus obstructing bone construction. SHP2’s capacity to dephosphorylate and galvanize JAK2 fosters JAK2-mediated signaling transduction. By triggering the NF-κB signaling avenue, regulating NFATC1 and c-fos expression, and influencing JAK2’s phosphorylation status, SHP2 supervises TAK1’s activities. Harmonizing with glucocorticoid receptors and RANKL, SHP2 ignites the SYK/SHP2/NF-κB cascade, thus endorsing osteoclastogenesis and propelling osteoporosis progression. SYK’s entanglement in bone resorption suggests its potential regulatory oversight over macrophages and other osseous cells, which might upset the homeostasis of bone remodeling, influencing bone density, and contributing to osteoporosis’s evolution. NF-κB’s mediation in inflammation, the proliferation and differentiation of bone cells, along bone remodeling, influence osteoporosis’s trajectory. An upsurge in the NF-κB signaling axis inhibits osteoblast development while proliferating osteoclast diversification. X-ray analyses showcasing a marked diminution in bone density in ovariectomized murines substantiate the osteoporosis model’s successful establishment. In ovariectomized mice, the targeted annulment of SHP2 expression within macrophages markedly bolsters bone density and ameliorates the osteoporotic state. The diminishment of SHP2 expression in these subjects noticeably curbs NF-κB and SHP2 levels, albeit not influencing SYK’s expression, underscoring SYK’s upstream positioning in the SHP2 pathway. In the BMMs from ovariectomized specimens, SHP2 gene silencing substantially curtails NFATC1, c-fos, and Cathepsin K expression, hence thwarting BMM transition to osteoclasts. The collaborative activation by glucocorticoid receptors and RANKL within the BMMs from ovariectomized mice, through the SYK/SHP2/NF-κB cascade, abets osteoclast formation and promotes swift osteoporosis onset [37–42].

Deploying ovariectomized mice as a foundational platform aid in the dissection of osteoporosis’s pathogenic mechanisms. Explicit SHP2 gene deactivation in macrophages, from these models, clarifies the therapeutic implications against osteoporotic manifestations, yielding empirical evidence for SHP2’s influence in osteoporosis. The revelation of glucocorticoid receptors and RANKL’s collaboration in instigating the SYK/SHP2/NF-κB signaling nexus thus potentiating osteoclast genesis, unveils fresh vantages on the mechanisms whereby glucocorticoids promote osteoporosis. Unveiling SHP2’s hand in modifying osteoblast differentiation, stimulating pathways for osteoclast inauguration, and participating in osteoporosis escalation renders novel enlightenment on SHP2’s operative schema in osteoporotic processes. To encapsulate, the potent symbiosis between glucocorticoids and RANKL energizes the SYK/SHP2/NF-κB pathway, endorsing bone marrow monocyte differentiation into osteoclasts and precipitating the advance of osteoporosis.
